# Supplementary material for: Inhibition of tau neuronal internalization using anti-tau single domain antibodies
Source: Nat Commun. 2025 Apr 2;16:3162. doi: 10.1038/s41467-025-58383-4 (PMC11965310; doi:10.1038/s41467-025-58383-4)
Supplement: Supplementary file 1 — Supplementary Information [file 41467_2025_58383_MOESM1_ESM.pdf]

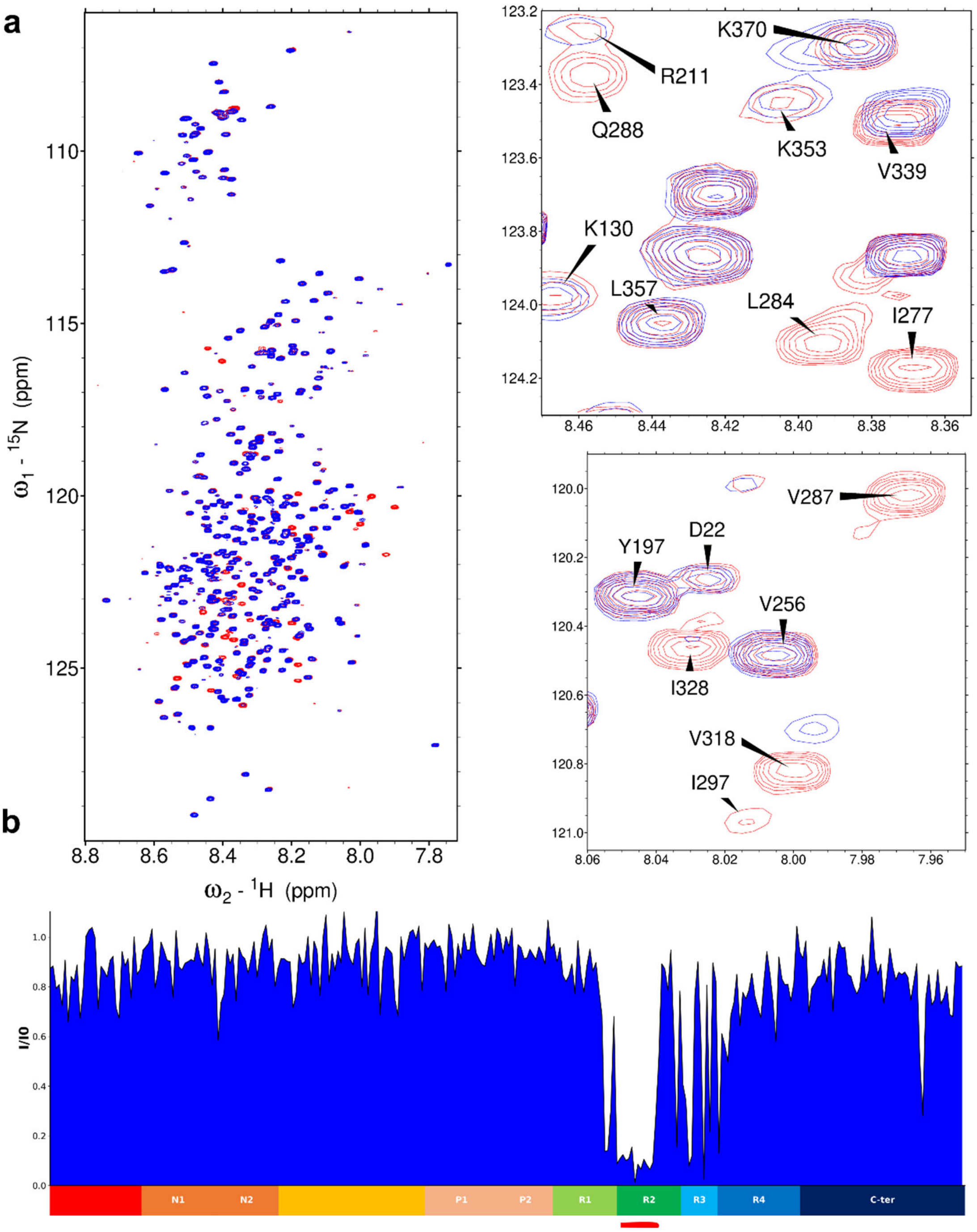

Supplementary Figure 1: VHH A31 binds to the R2 repeat domain of tau.
a. Overlay of ^{1}H, ^{15}N HSQC two-dimensional full spectra and details of free full-length tau2N4R (in red) or tau2N4R mixed with unlabeled VHH A31 (in blue) (the spectrum is representative of n = 2 independent experiments). In the spectrum of tau in the presence of VHH A31, several resonances are broadened beyond detection compared to the tau control spectrum. **b**. Normalized NMR intensities (I/I0) along the tau sequence, where (I0) and (I) correspond to the resonance intensity when tau is free in solution or mixed with an equimolar amount of VHH A31 (I), respectively. The normalized intensity ratio plot (I/I0) allowed the identification of the tau repeat 2 domain as the target of VHH A31 interaction. A red line indicates the region containing the corresponding major broadened resonances, which was mapped to the R2 domain containing the PHF6* sequence. N1 and N2 are two inserted sequences in the N-terminal domain that are not present in all Tau isoforms (named tau 0N, tau 1N or tau 2N), the proline-rich domain is subdivided in P1 and P2 regions, the MTBD consists of four partially repeated regions, R1 to R4.

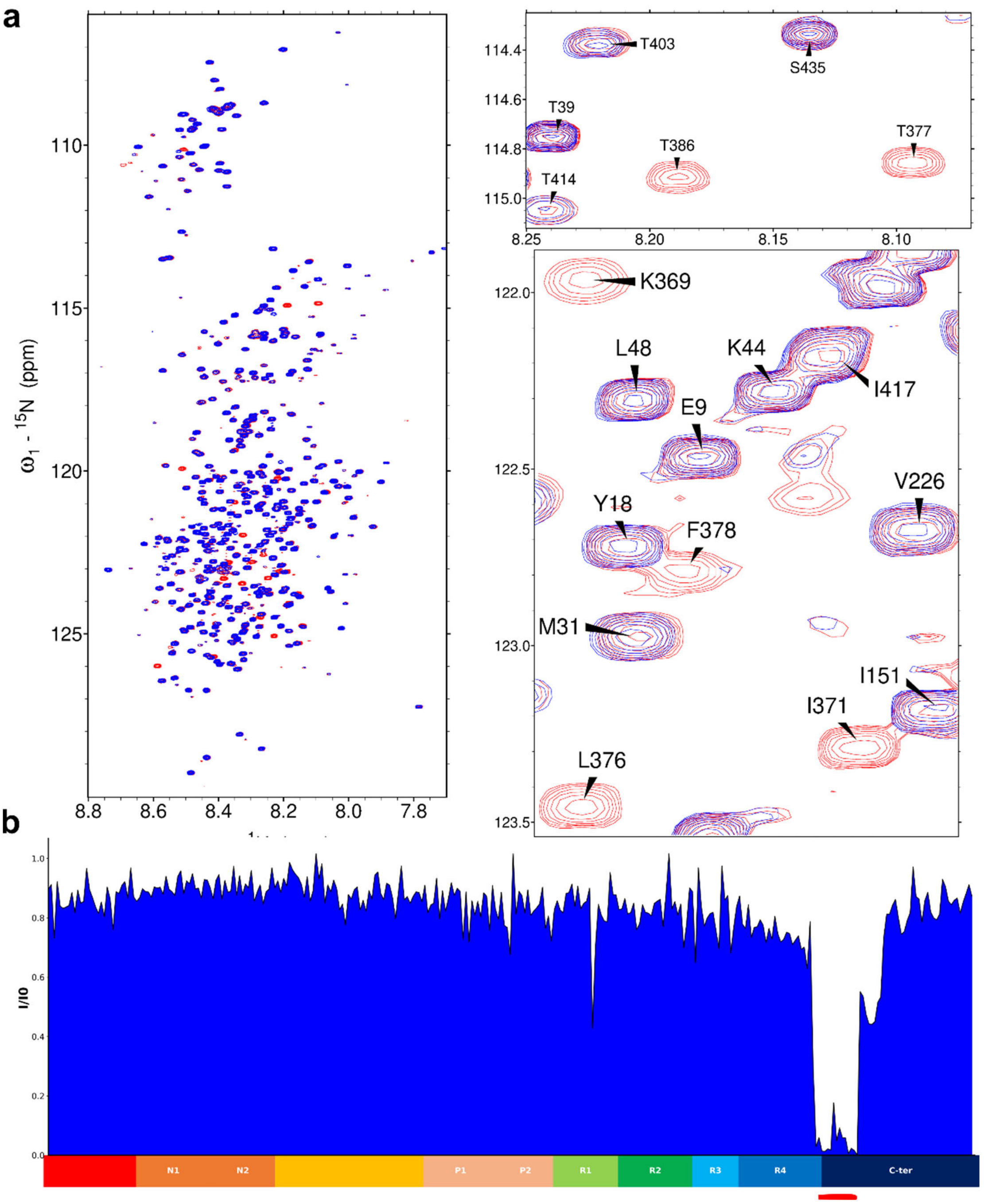

Supplementary Figure 2: VHH E2-2 binds to the C-terminal domain of tau.

a. Overlay of ^{1}H, ^{15}N HSQC two-dimensional full spectra and details of free full-length tau2N4R (in red) or tau2N4R mixed with unlabeled VHH E2-2 (in blue) (the spectrum is representative of n = 2 independent experiments). In the spectrum of tau in the presence of VHH E2-2, several resonances are broadened beyond detection compared to the tau control spectrum. **b**. Normalized NMR intensities (I/I0) along the tau sequence where (I0) and (I) correspond to the resonance intensity when tau is free in solution or mixed with an equimolar amount of VHH E2-2 (I), respectively. The normalized intensity ratio plot (I/I0) allowed the identification of the tau C-terminal domain as the target of the VHH E2-2 interaction. A red line indicates the region containing the corresponding broadened resonances, which was mapped to the C-terminal sequence, following the repeat region R4.

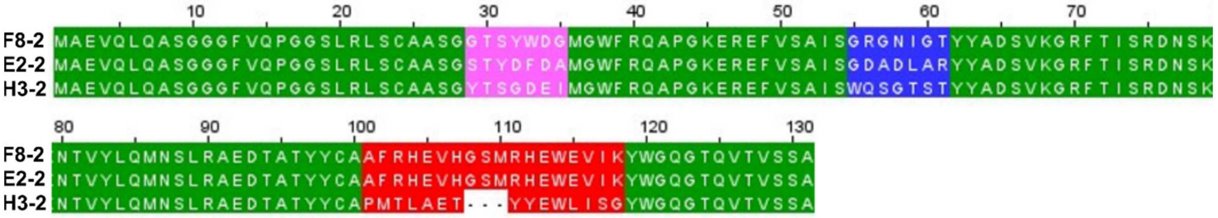

Supplementary Figure 3: VHHs F8-2 and E2-2 retain the same CDR3 recognition loop.
Sequence alignment of VHHs F8-2, E2-2 and H3-2, showing conservation of the CDR3 loop sequence (in red) for F8-2 and E2-2, but not for the CDR1 and CDR2 loops (in pink and blue, respectively).

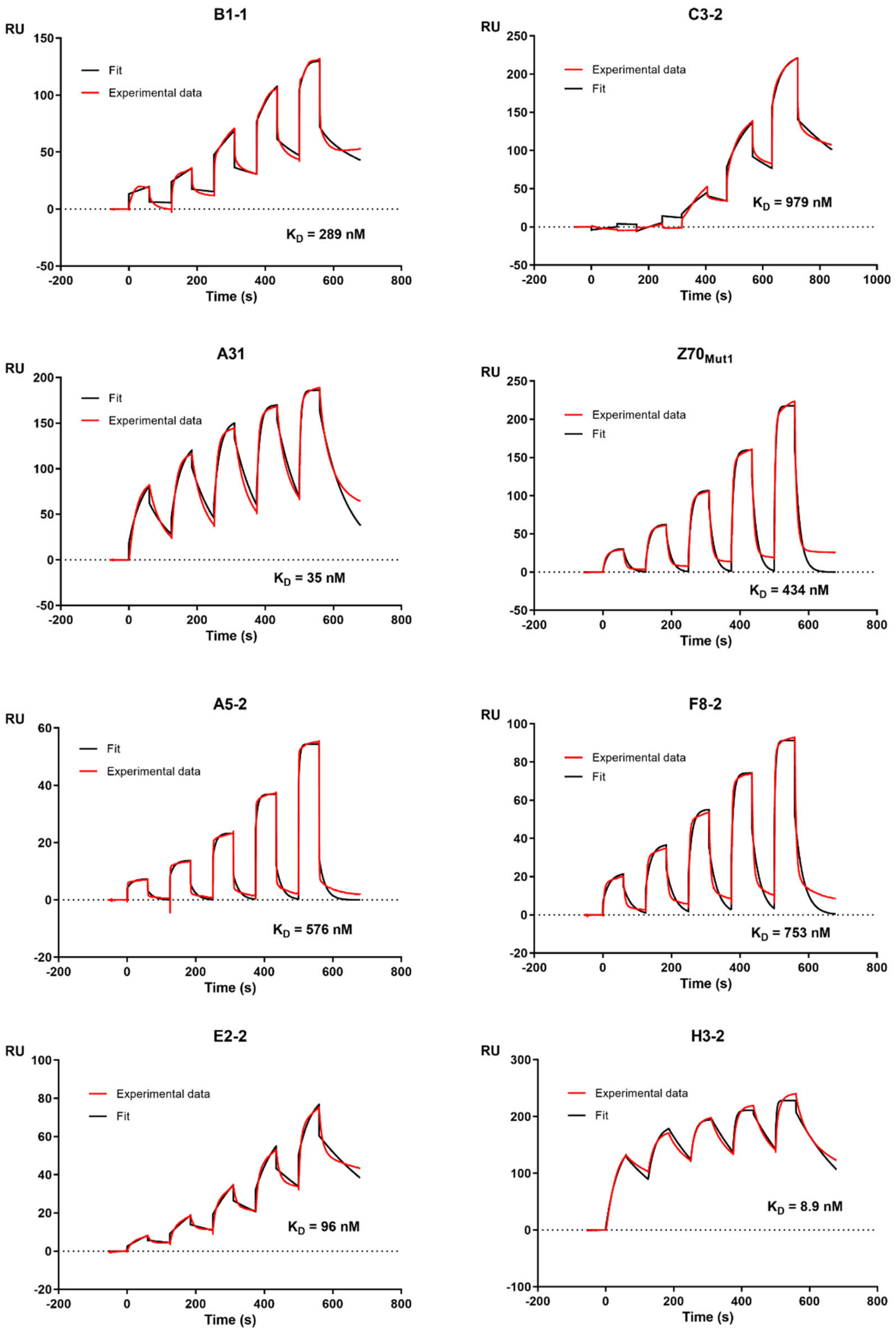

Supplementary Figure 4: Affinity determination for the series of VHHs binding tau2N4R by SPR. Sensorgrams (reference subtracted data) of single cycle kinetics analysis performed on immobilized biotinylated tau2N4R, with five injections of increasing concentrations of VHH B1-1, C3-2, A31, $Z70_{mut1}$, A5-2, F8-2, E2-2 and H3-2 (sensorgrams are representative of a n = 2 independent experiments). k_{on} and k_{off} and K_D values are included in Table 1. Black lines correspond to the fitted curves, red lines correspond to the measurements.

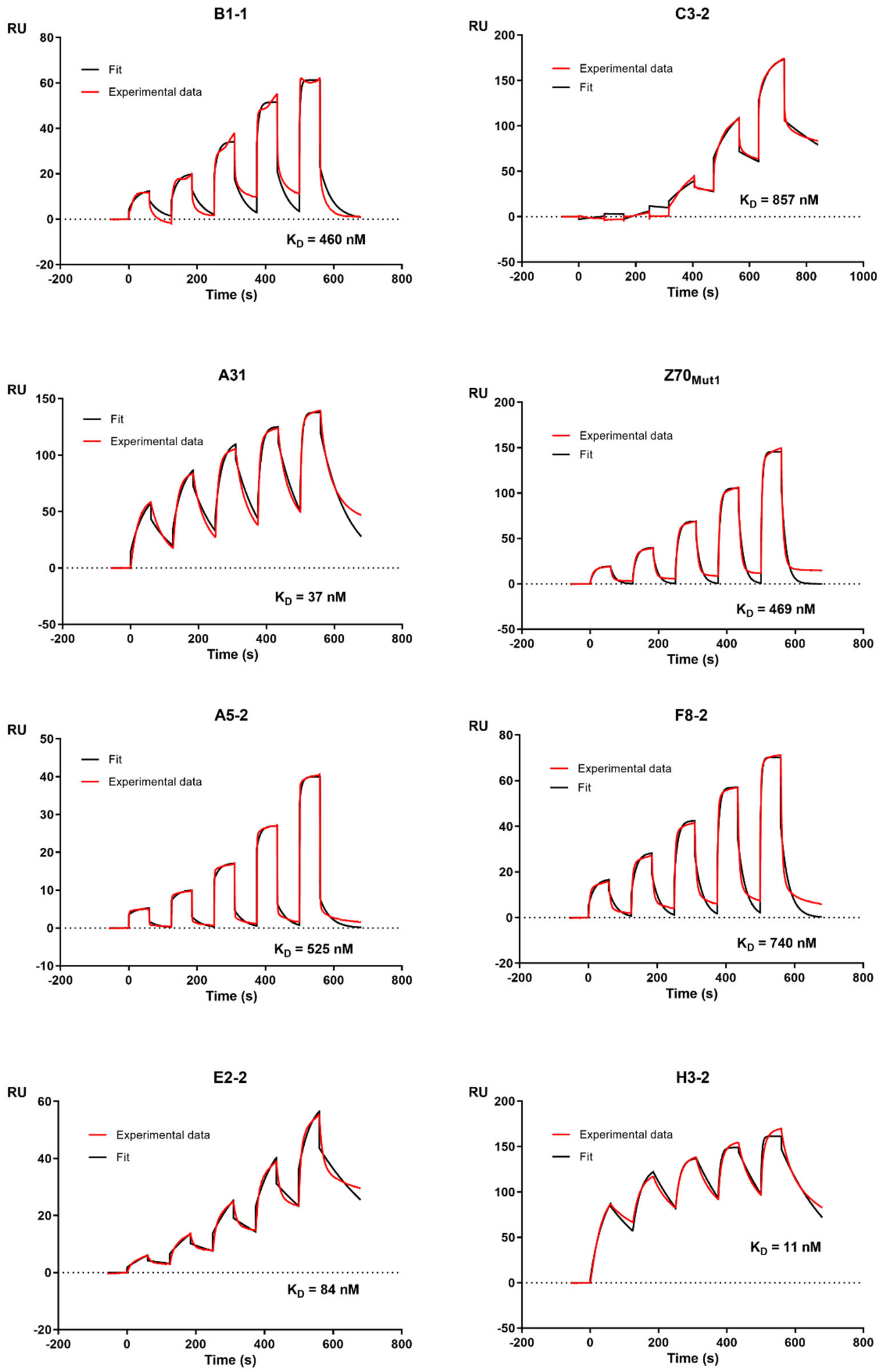

Supplementary Figure 5: Affinity determination for the series of VHHs binding tau1N4R by SPR. Sensorgrams (reference subtracted data) of single cycle kinetics analysis performed on immobilized biotinylated tau1N4R, with five injections of increasing concentrations of VHH B1-1, C3-2, A31, Z70$_{mut1}$, A5-2, F8-2, E2-2 and H3-2 (sensorgrams are representative of a n = 2 independent experiments). k_{on} and k_{off} and K_D values are included in Table 1. Black lines correspond to the fitted curves, red lines correspond to the measurements.

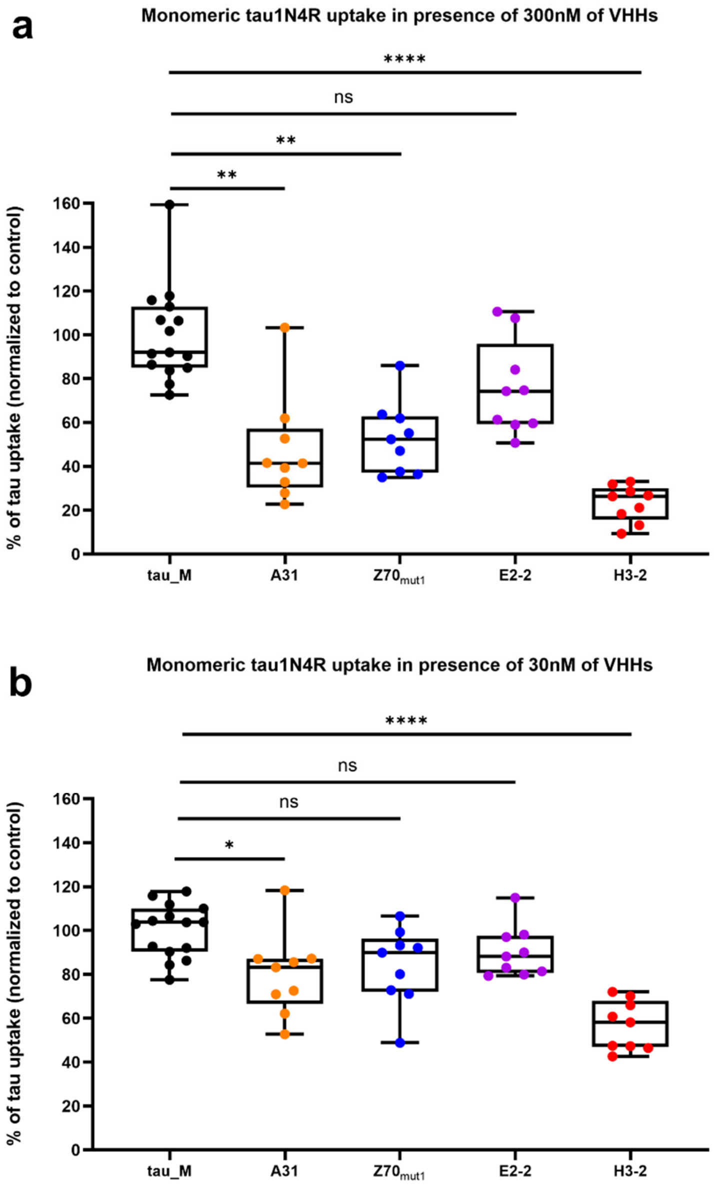

Supplementary Figure 6: Tau cellular internalization assay using 30 and 300 nM of VHHs
a. Percentage of cellular tau uptake based on Alexa546 fluorescence signal coming from tau co-incubated with each VHH at a concentration of 30 nM (n = 3 independent experiments per condition, technical triplicate per experiment). **b**. Percentage of cellular tau uptake based on Alexa546 fluorescence signal coming from tau co-incubated with each VHH at a concentration of 300 nM (n = 3 independent experiments per condition, technical duplicate or triplicate per experiment). Data were normalized to the condition of tau1N4R pre-incubated with PBS (tau_M) as 100% of tau uptake. Box plots indicate median (middle line), 25th, 75th percentile (box) and minimum and maximum values (whiskers), all data points are shown (single points). Data were analyzed using one-way nonparametric ANOVA (Kruskal-Wallis) with Dunn's multiple comparison test (****$p < 0.0001$;***$p < 0.001$; **$p < 0.01$; *$p < 0.05$; ns : non significant). VHHs A31, and H3-2 significantly reduced cellular tau uptake at a concentration of 30 nM. VHHs A31, $Z70_{mut1}$, and H3-2 significantly reduced cellular tau uptake at a concentration of 300 nM.

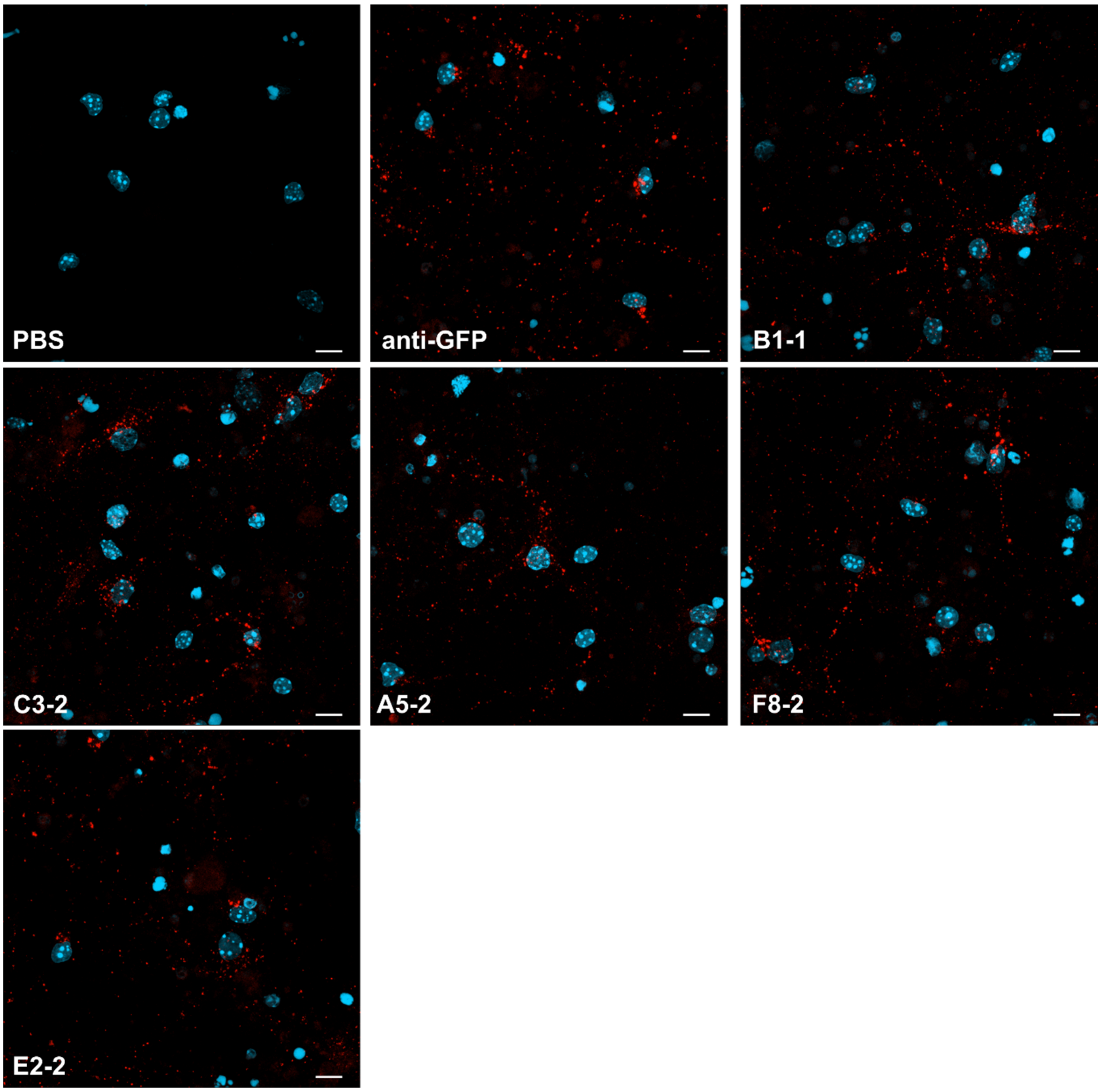

Supplementary Figure 7: Confocal microscopy visualization of cellular internalization of monomeric tau1N4R in primary neuronal cultures in the presence of the anti-tau VHHs

Confocal microscopy analysis of cellular tau uptake assay using PBS, 50 nM of monomeric tau1N4R labeled with Alexa546 dye and in the presence of 100 nM final of VHH anti-GFP, B1-1, C3-2, A5-2, F8-2 or E2-2 (Images are representative of a n = 2 independent experiments per condition). Tau was visualized in red and nuclei in light blue. Scale bar is 10 µm.

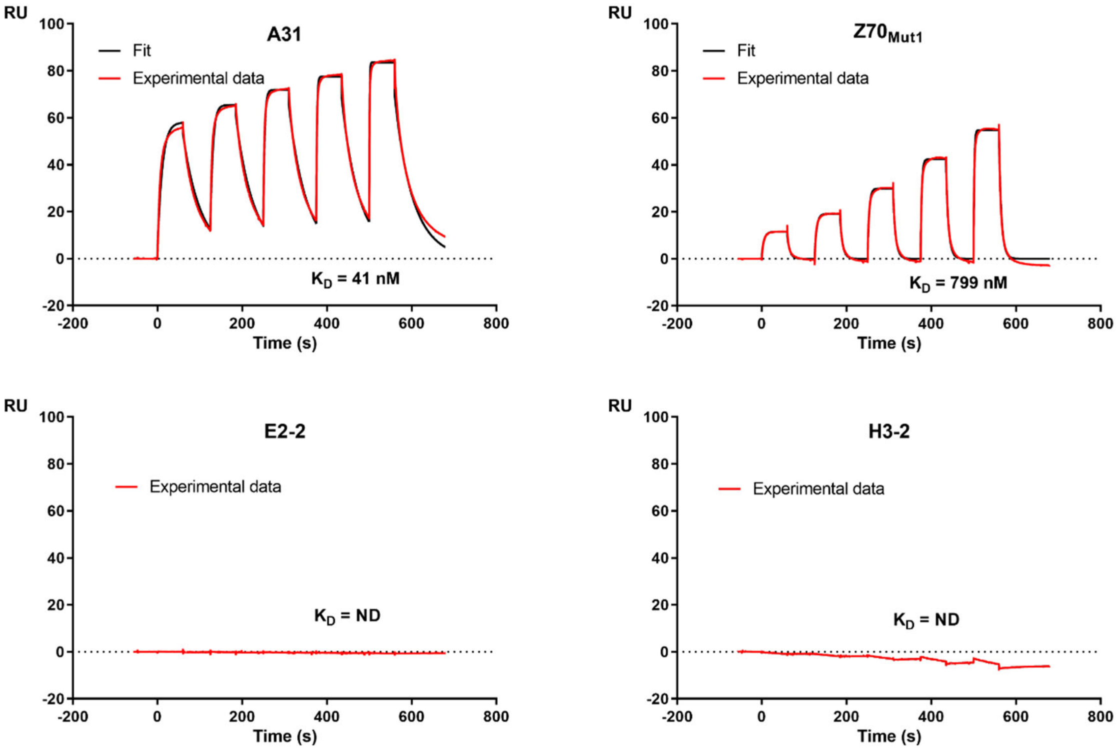

VHH	k_{on} $(M^{-1}.s^{-1})*10^{+2}$	k_{off} $(s^{-1})*10^{-3}$	K_D (nM)
A31	6077 ± 63	24.8 ± 0.2	41 ± 1
$Z70_{mut1}$	2605 ± 59	208 ± 5.0	799 ± 27

Supplementary Figure 8: Affinity determination for the series of VHHs binding to tauMTBD by SPR. Sensorgrams (reference subtracted data) of single cycle kinetics analysis performed on immobilized biotinylated tauMTBD, with five injections of VHH A31, $Z70_{mut1}$, E2-2 and H3-2 at 0.125 µM, 0.25 µM, 0.5 µM, 1 µM, and 2 µM (sensorgrams are representative of a n = 2 independent experiments). Black curves correspond to the fitted curves, red curves correspond to the measurements. k_{on} and k_{off} and K_D values are given in the table below.

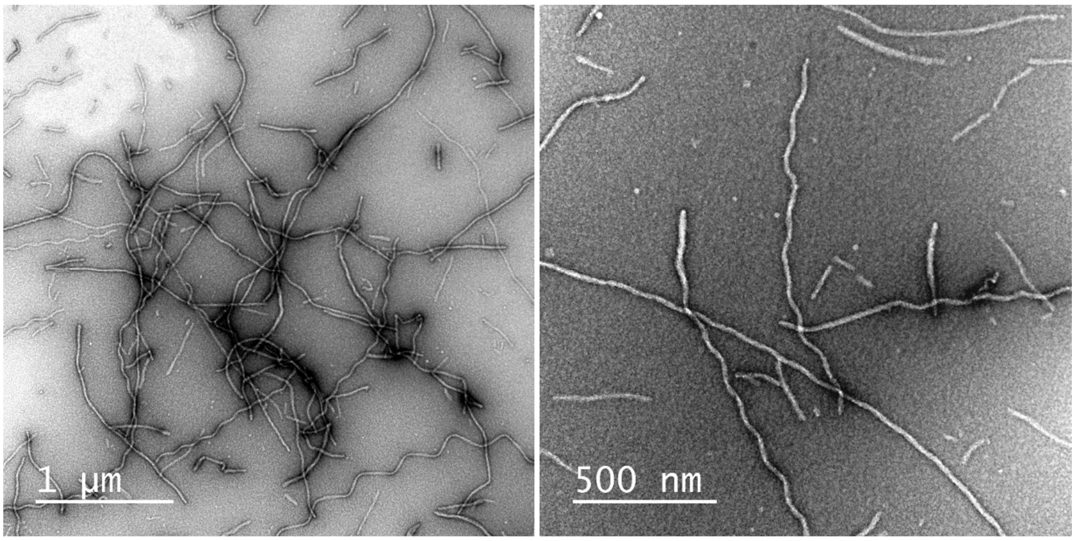

Supplementary Figure 9: Visualization of labeled tau1N4R fibrils
Transmission electron microscopy images at the end point of the aggregation assay of labeled Atto565-tau1N4R.

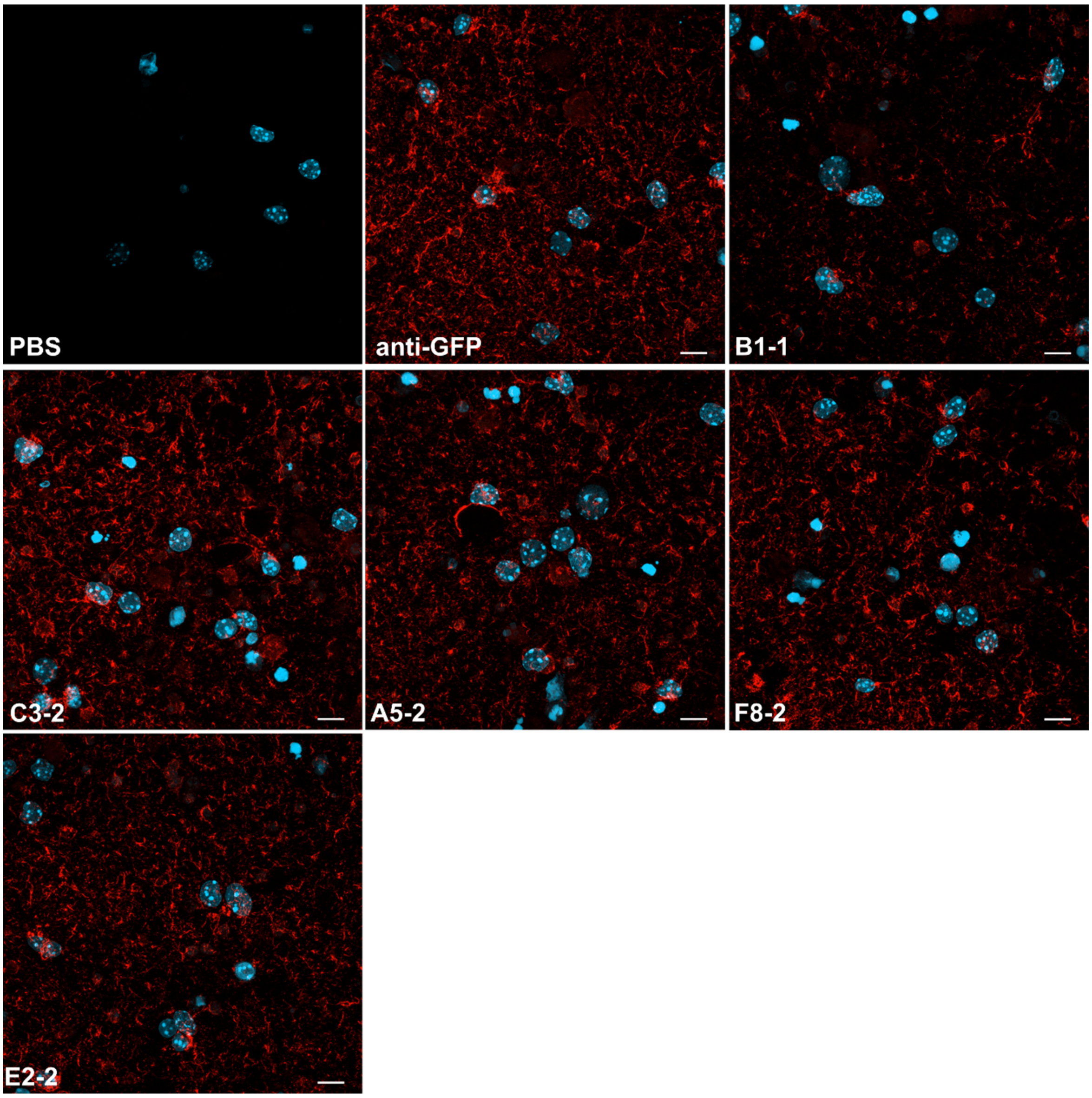

Supplementary Figure 10: Confocal microscopy visualization of cellular internalization of tau1N4R fibrils in primary neuronal cultures in the presence of the anti-tau VHHs
Confocal microscopy analysis of cellular tau fibril uptake assay using PBS, 200 nM of tau1N4R fibrils labeled with Atto565 dye and in the presence of 400 nM final of VHH anti-GFP, B1-1, C3-2, A5-2, F8-2 or E2-2 (images are representative of a n = 2 independent experiments per condition). Tau was visualized in red and nuclei in light blue. Scale bar is 10 µm.

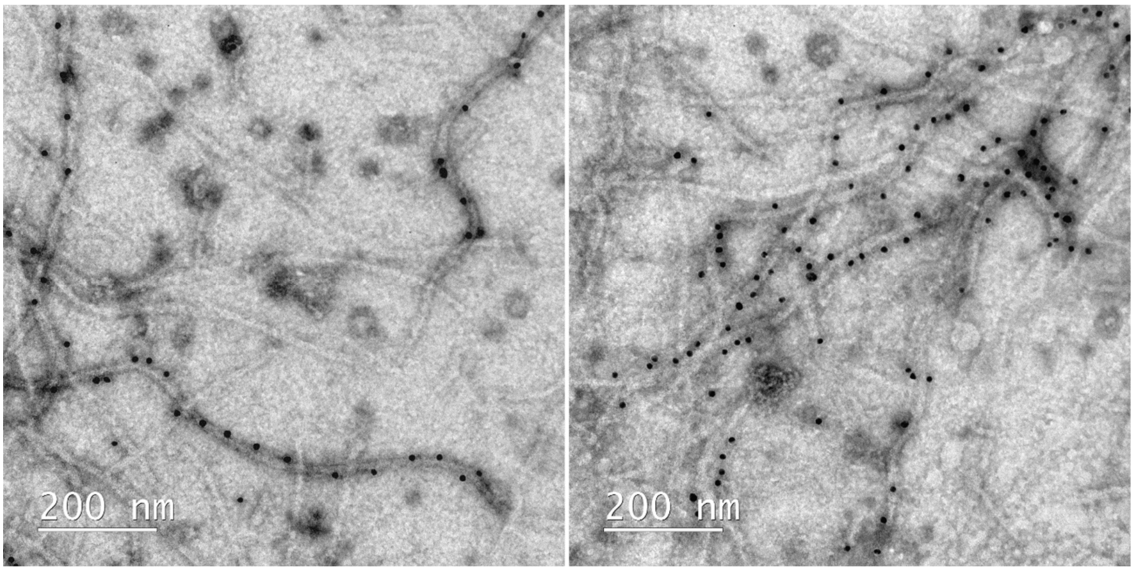

Supplementary Figure 11: VHH H3-2 binds to recombinant tau2N4R fibrils
Transmission electron microscopy images showing H3-2-gold nanoparticles interacting with tau2N4R fibrils.

a

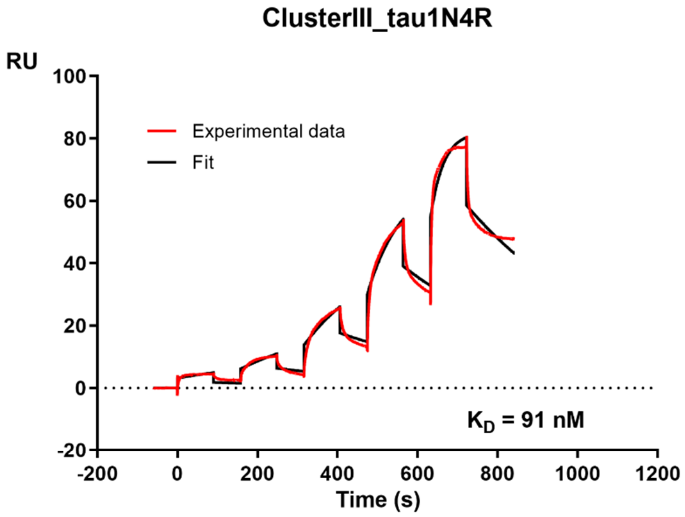

Protein	k_{on} $(M^{-1}.s^{-1})*10^{+2}$	k_{off} $(s^{-1})*10^{-3}$	K_D (nM)
tau1N4R	280 ± 1.4	2.6 ± 0.03	91 ± 1

b

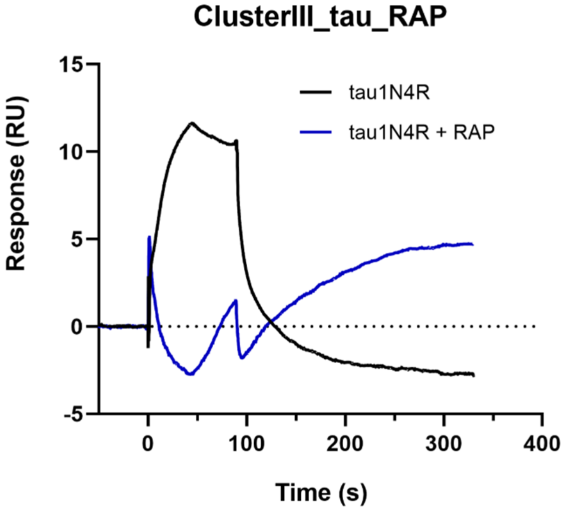

Supplementary Figure 12: Affinity determination between tau and LRP1 cluster III and competition experiment between tau1N4R and human RAP on LRP1 cluster III by SPR
a. Sensorgram (reference subtracted data) of single cycle kinetics analysis performed on immobilized LRP1 cluster 3 Fc-tagged protein, with five injections of tau1N4R at 0.012 µM, 0.037 µM, 0.11 µM, 0.33 µM, and 1 µM (sensorgrams are representative of a n = 2 independent experiments). k_{on} and k_{off} and K_D values are included in the table. Black curves correspond to the fitted curves, red curves correspond to the measurements. **b**. Sensorgram (control subtracted data) of tau1N4R binding to immobilized LRP1 cluster III in the absence or presence of 0.3 µM of human RAP protein.

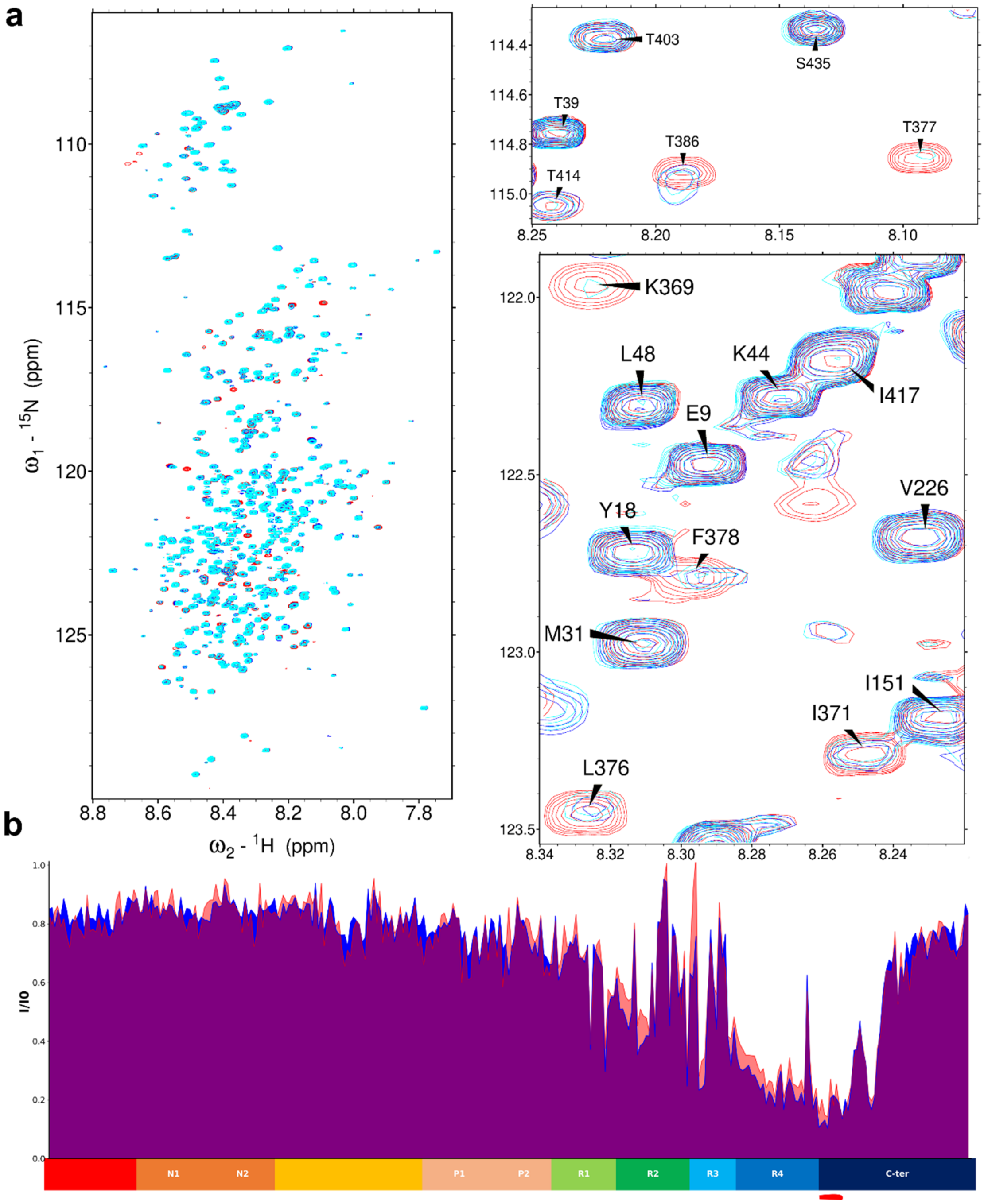

Supplementary Figure 13: Tau MTBD do not compete with full-length tau2N4R-H3-2 binding.
a. Overlay of ^{1}H, ^{15}N HSQC two-dimensional full spectra and details of free 100 µM of full-length tau2N4R (in red), 100 µM of tau2N4R mixed with 100 µM of unlabeled VHH H3-2 (in blue) or 100 µM tau2N4R mixed with 100 µM of unlabeled VHH H3-2 and 400 µM of unlabeled tauMTBD (in cyan blue). In the spectrum of tau in the presence of VHH H3-2 or H3-2-tauMTBD, the same resonances are broadened beyond detection compared to the tau control spectrum. **b**. Normalized NMR intensities (I/I0) along the tau sequence where (I0) corresponds to the resonance intensity when tau is free in solution and (I) corresponds to the resonance intensity when tau is mixed with an equimolar amount of VHH H3-2 (blue) or mixed with an equimolar amount of VHH H3-2 and 4-fold amount of tauMTBD (light red). The normalized intensity ratio plot (I/I0) allowed the identification of the tau C-terminal domain as the target of the VHH H3-2 interaction and showed that tauMTBD did not affect the binding of VHH H3-2 to full-length tau. A red line indicates the region containing the corresponding broadened resonances, which was mapped to the C-terminal sequence.

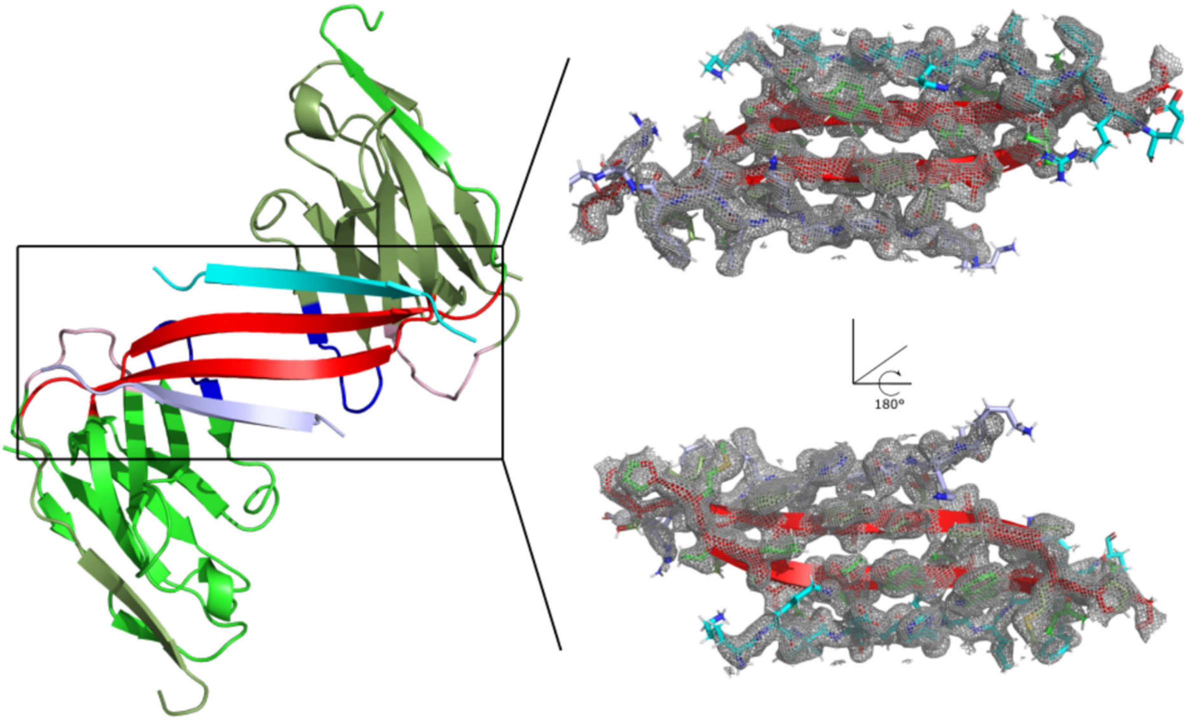

Supplementary Figure 14: Crystal structure of VHH H3-2 in complex with the C-ter peptide.
Left: Cartoon representation of a H3-2 dimer with one monomer in light green and the other in dark green with their CDR1 colored in pink, CDR2 in blue and CDR3 in red. The interacting peptides are shown in cyan and light blue. Right: Electron density (2fo-fc map contoured at 1 σ), shown as mesh representation, corresponding to the CDR3 and peptide residues, with 180° rotation along the x-axis.

Supplementary Table 1 : Crystallography data collection and refinement statistics

**pdb9G13**	
Wavelength	0.9786A
Resolution range	38.17 - 1.80 (1.864 - 1.800)
Space group	P 21 21 21
Unit cell	70.39 76.34 95.56 90 90 90
Total reflections	617379 (59698)
Unique reflections	48399 (4759)
Multiplicity	12.8 (12.5)
Completeness (%)	99.89 (99.71)
Mean I/sigma(I)	26.2 (3.4)
Wilson B-factor (Å²)	29
R-merge	0.278 (1.284)
R-meas	0.289 (1.338)
R-pim	0.080 (0.373)
CC1/2	0.999 (0.869)
CC*	1 (0.964)
Reflections used in refinement	48373 (4750)
Reflections used for R-free	2347 (235)
R-work	0.177 (0.309)
R-free	0.232 (0.347)
CC(work)	0.964 (0.878)
CC(free)	0.930 (0.722)
Number of non-hydrogen atoms	4716
macromolecules	4246
ligands	0
solvent	470
Protein residues	551
RMS Bond lengths (Å)	0.016
RMS Bond angles (°)	2.44
Ramachandran favored (%)	93.83
Ramachandran allowed (%)	4.30
Ramachandran outliers (%)	1.87
Rotamer outliers (%)	3.01
Clashscore	6.17
Average B-factor (Å²)	39
macromolecules	39
solvent	44
VHHs	38
peptides	48

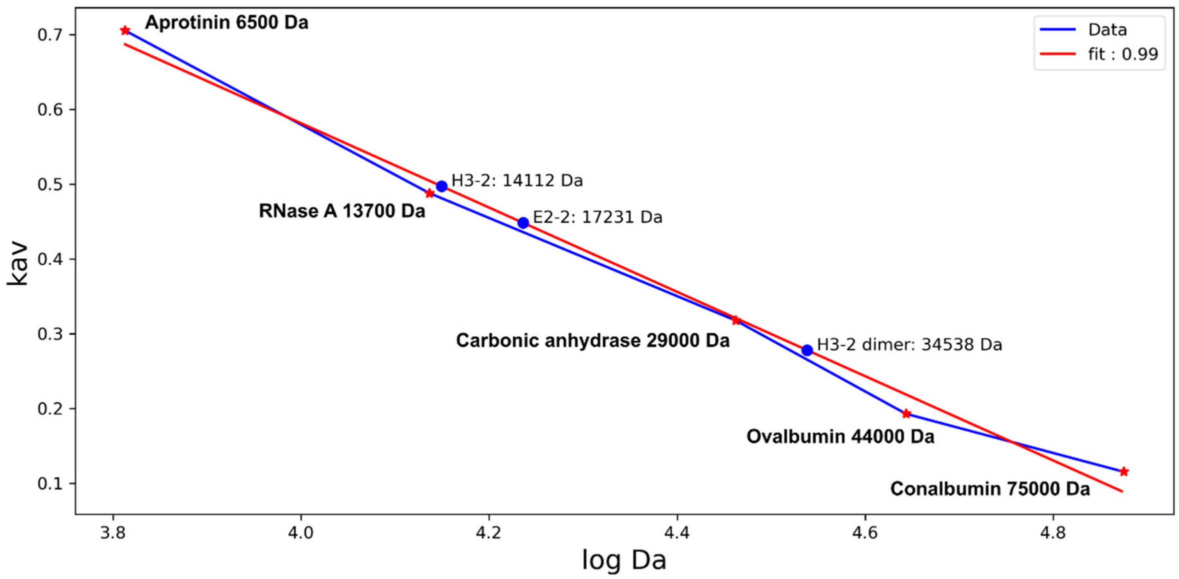

Supplementary Figure 15: Calibration curve and molecular weight estimation of VHHs H3-2 and E2-2 in the absence or in presence of tau C-terminal peptide.
Calibration curve recapitulating the size exclusion chromatography (SEC) analysis of five injected proteins of different and known molecular weights (red stars) and that have been used to fit and estimate the molecular weights of VHH H3-2 and E2-2 (red curve, blue dots). Log Da is the logarithm of the molecular weights in daltons. K_{av} is calculated as V_e-V_0/V_t-V_0 where V_e is the elution volume, Vt is the total volume, and V_0 is the exclusion volume of the a Superdex^{TM} 75 Increase 10/300 GL column.

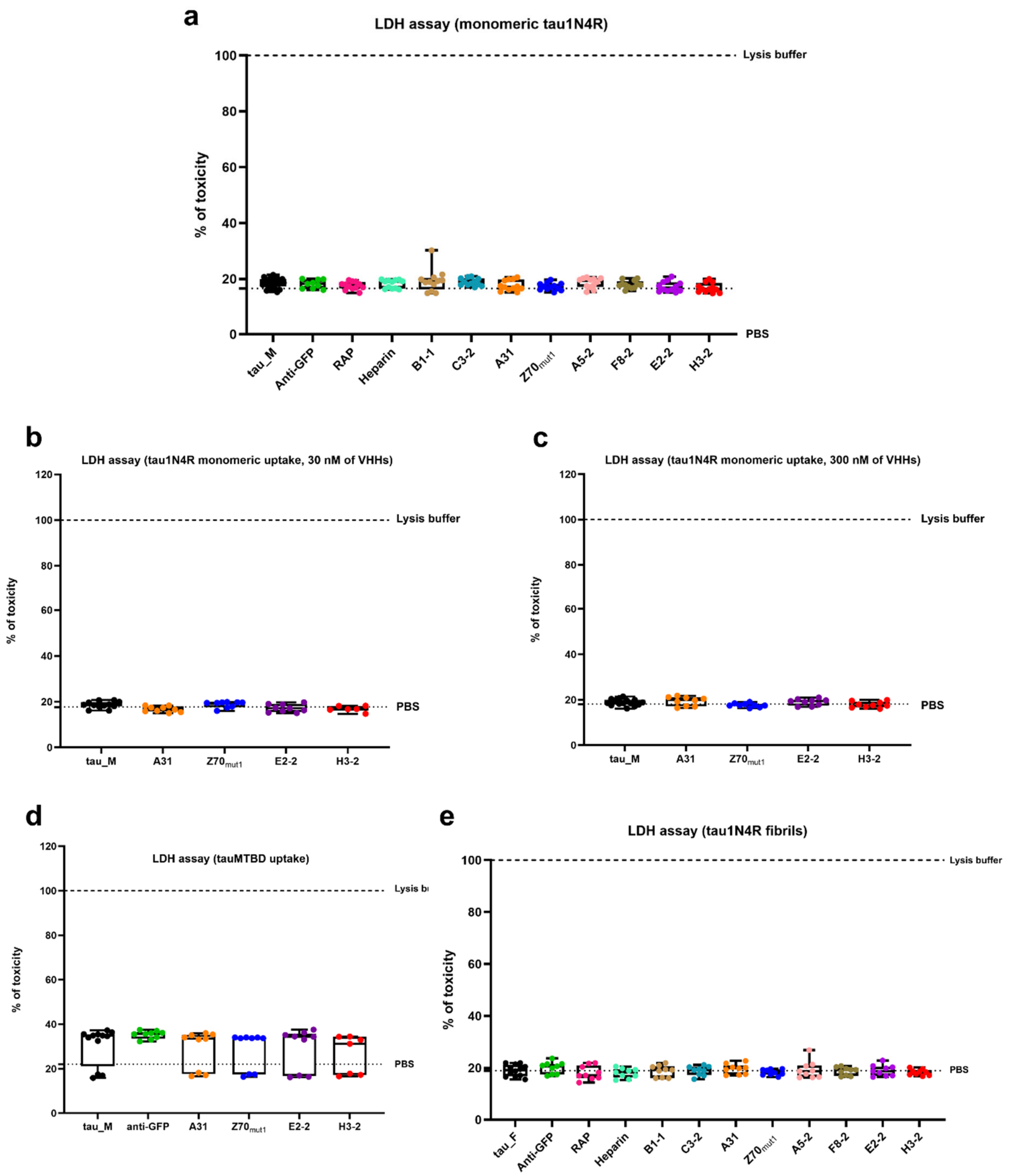

Supplementary Figure 16: Tau-VHH co-incubation do not induce cellular toxicity.
Results of the lactate dehydrogenase (LDH) assay performed on the primary neuronal culture after incubation with 50 nM of tau1N4R in PBS (tau_M) or co-incubated with the controls (RAP or heparin) or the VHHs at a final concentration of **a.** 100 nM (n = 4 independent experiments, technical duplicate or triplicate per experiment), **b**. 30 nM or **c**. 300 nM (n = 3 independent experiments, duplicate or triplicate per experiment). **d**. Results of the lactate dehydrogenase (LDH) assay performed on the primary neuronal culture after incubation of the tauMTBD-VHH complexes (n = 3 independent experiments, technical duplicate or triplicate per experiment). **e**. Results of the lactate dehydrogenase (LDH) assay performed on the primary neuronal culture after incubation of 200 nM of tau1N4R fibrils in PBS (tau_M) or co-incubated with the controls (RAP or heparin) or the VHHs at a final concentration of 400 nM (n = 3 independent experiments, technical duplicate or triplicate per experiment). Data were normalized to 100% toxicity corresponding to cell lysis (dashed horizontal line). The PBS condition corresponds to the LDH assay results performed on the neuronal culture that have been incubated with PBS buffer only (dashed horizontal line). Box plots indicate median (middle line), 25th, 75th percentile (box) and minimum and maximum values (whiskers), all data points are shown (single points).
